# Factors associated with newborn care knowledge and practices in the upper Himalayas

**DOI:** 10.1371/journal.pone.0222582

**Published:** 2019-09-16

**Authors:** Devendra Raj Singh, Chloe M. Harvey, Pushpalata Bohara, Dhirendra Nath, Sunita Singh, Sylvia Szabo, Kshitij Karki

**Affiliations:** 1 Department of Public Health, Asian College for Advance Studies, Purbanchal University, Lalitpur, Nepal; 2 Research and Innovation Section, Southeast Asia Development Actions Network (SADAN), Lalitpur, Nepal; 3 Department of Social Statistics and Demography, University of Southampton, Southampton, England, United Kingdom; 4 Sukraraj Tropical & Infectious Disease Hospital, Ministry of Health & Population, Kathmandu, Nepal; 5 Department of Development and Sustainability, Asian Institute of Technology, Pathum Thani, Thailand; 6 Program and Research Department, Group for Technical Assistance, Lalitpur, Nepal; Liverpool School of Tropical Medicine, UNITED KINGDOM

## Abstract

**Background:**

Globally, neonatal deaths remain a major public health challenge and account for the majority of deaths occurring among children under five years of age. Despite Nepal’s significant achievements in meeting the maternal and child health targets of the Millennium Development Goals, an estimated 23,000 Nepalese children under five years die every year, with three out of five babies dying within the first 28 days of life. This study therefore aimed to examine the level of knowledge and practices of newborn care among Nepalese mothers in the upper Himalayas and the factors associated with these.

**Materials and methods:**

A community based cross-sectional study was conducted among 302 randomly selected mothers with children under two years of age in Tripurasundari Municipality of Dolpa district, an upper Himalayan region of Nepal. Mothers were interviewed using semi-structured questionnaires. Mean score for knowledge and Bloom’s criteria for practice were considered to categorize newborn care knowledge and practices. Multivariate logistic regression was used to identify factors associated with the newborn care knowledge and practices.

**Results:**

In this study, 147 (48.7%) of the mothers were found to have inadequate knowledge of newborn care, while 102 (33.8%) mothers had reported unsatisfactory newborn care practices. Mothers with at least secondary level of formal education were more likely to possess adequate newborn care knowledge compared to mothers who never attended school (AOR 4.93 at 95% CI 1.82–13.33). Mothers whose first pregnancy occurred between the ages of 20–24 years (AOR 3.89 at 95% CI 1.81–8.37) were also more likely to possess adequate newborn care knowledge, compared to mothers with a younger age at first pregnancy. Furthermore, mothers who had completed at least four ANC visits (AOR 2.89 at 95% CI 1.04–7.96), mothers who had completed three PNC visits (AOR 2.79 at 95% CI 1.16–6.72) and mothers who reported that their nearest health facility was less than one hour (30–59 minutes) walking distance (AOR 3.66 at 95% CI 1.43–9.33) had higher odds of having adequate newborn care knowledge. Similarly, mothers whose household monthly income was more than $100 (AOR 4.17 at 95% CI 1.75–9.69), mothers who had completed three PNC visits (AOR 3.27 at 95% CI 1.16–9.20) and mothers with adequate newborn care knowledge (AOR 15.35 at 95% CI 5.82–40.47) were found to be more likely to practice a satisfactory level of newborn care practices in adjusted analysis.

**Conclusion:**

The study revealed high prevalence of inadequate newborn care and knowledge amongst mothers in upper Himalayan dwellings. Approximately one third of all interviewed mothers practiced suboptimal newborn care. The results indicate an urgent need to increase awareness of neonatal services available to mothers and to prioritize investments by local governments in neonatal health services, in order to improve accessibility and quality of care for mothers and newborns.

## Background

Globally, neonatal mortality remains a major public health challenge and accounts for approximately half of deaths occurring among children under five years of age[1]. The majority of these neonatal deaths occur in the first seven days of life due to preventable medical conditions [2,3]. In 2016 alone, 2.6 million children died in the first month of life, an estimated 7,000 neonatal deaths occurring every day worldwide[1,3]. Approximately 99% of neonatal deaths occur in resource poor settings [4] which equates to a mortality risk which is six times higher for babies born in developing countries compared to those born in developed countries [5]. Preterm birth, severe infections, asphyxia and neonatal tetanus are the major direct causes of global neonatal deaths [4]. Therefore, effective implementation of World Health Organization (WHO) recommendations for essential newborn care is key to newborn survival and includes early initiation of breast-feeding, cord care, eye care, thermoregulation, management of asphyxia, recognition of neonate danger signs, immunization and care of low birth weight neonates[6].

Although Nepal is one of the few developing nations to achieve all targets under Millennium Development Goal (MDG) 4 [7], an estimated 23,000 Nepalese children under-five years of age still die every year, with three out of five newborns dying within the first 28 days of life[8]. According to the most recent 2016 Nepal Demographic and Health Survey (NDHS), the neonatal mortality rate decreased at a much slower pace to 21 deaths per 1,000 live births [9]. The geographical differences in neonatal deaths range from 15 to 41 neonate deaths per 1,000 live births[9]. Due to the fact that many geographical areas in Nepal are hard to reach, the government has implemented community-based service delivery approaches such as the Community-Based Newborn Care Program (CB-NCP) in 2007 and a Community Based Integrated Management of Newborn and Childhood Illness (CB-IMNCI) in 2015 to improve child health across the country. [10]. To further accelerate the progress, the Government of Nepal has also recently endorsed Nepal’s Every Newborn Action Plan-2016 (NENAP) with the objective to implement cost effective and evidence based newborn care to meet SDG target 3.2 [11].

Indicators of child health vary extensively across different provinces and ecological zones in Nepal [12]. In a developing context such as Nepal, it is important to identify the socio-demographic factors and maternal health practices associated with neonatal mortality, in order to target child health interventions at mothers and children who are considered at risk. In Nepal, knowledge of newborn care and use of health facilities varies between the geographical and ecological regions, due to the social and cultural belief systems of different ethnic groups and inequalities in health care coverage[9,12–14]. Forty one percent of total deliveries occur at home, often in an animal shed or separate, congested room, which exacerbates the risk of infection transmission to both mother and newborn[9,15,16]. Moreover, several studies have highlighted poor child health status and elevated neonatal mortality in the upper Himalaya region of Nepal, despite the implementation of several newborn and maternal health policies by the government[8,9,17]. However, very few studies thus far have explored maternal newborn care knowledge and variations in practices by different ecological regions in Nepal. The objectives of this study were (1) to examine knowledge on newborn care among mothers and practices on newborn care; and (2) to identify the factors associated with newborn care knowledge and practices in Tripurasundari Municipality of Dolpa district, an upper Himalayan region.

## Materials and methods

### Study design and setting

A community based cross-sectional study was conducted between November 2017 and February 2018, in Dolpa district in the upper Himalayan region of Nepal; situated in Karnali province with an altitude of over 5000 meters. The district is bordered by China on the North and has very limited roadway links, infrastructure and health care facilities. The district is an example of extreme remoteness with very low literacy, poor socio-economic status, high prevalence of child marriage, high prevalence of child malnutrition, low life expectancy, low institutional delivery, and limited access to basic facilities among the majority of the population[9,17].

**Fig 1:**
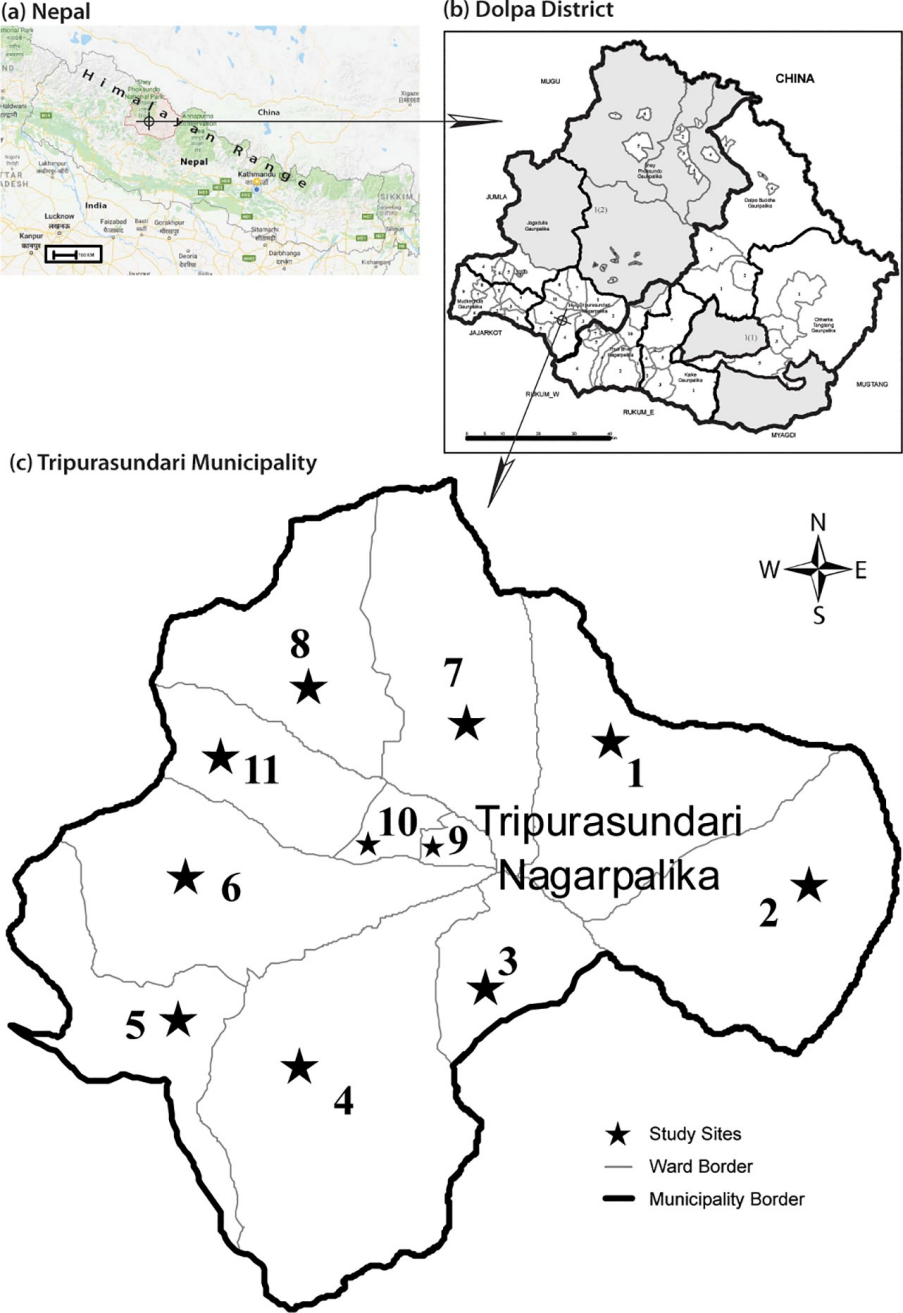
Location of Tripurasundari Municipality. The figure shows the location of study sites (a) Nepal. (b) Dolpa District. (c) Tripurasundari Municipality.

Dolpa district is divided into two municipalities and six rural municipalities. Tripurasundari municipality was randomly selected for the study site (Fig 1**)**. According to the National population and housing census 2011, conducted by the Central Bureau of Statistics, Tripurasundari had a total population of 10,104 (4966 males and 5138 females) of which the population of reproductive age (15–49 years) was 6604 (4452 males and 2,152 females)[18].

### Sample and exclusion criteria

Sample size was calculated by using single population proportion formula for finite population (*n* = [Np(1-p)]/ [(d^2^/Z^2^_1-α/2_*(N-1)+p*(1-p)]). A total of 2,152 female population of reproductive age group (15–49) were used as the finite population (N) [18].The proportion (p) was considered 69% i.e. using antibiotic ointment (Chlorhexidine) applied on the stump of the umbilical cord within an hour of cord being cut [9]; absolute precision (d) of 5%, and standard normal deviation (Z) at confidence limit of 95%. The sample was adjusted to 314 with addition of 10% non-response rate to the calculated sample size.

Mothers with at least one child aged less than two years old were considered eligible participants for this study. Mothers who were admitted to a health facility for delivery, those with hearing and speech disabilities, and who were not present at home on the day of data collection were excluded from the study.

A systematic random sampling method was applied to select the required sample from a list of households with mothers who had at least one child younger than two years old. This list was obtained from local Female Community Health Volunteers (FCHVs). Within each local administrative unit, there is at least one FCHV, who work voluntarily to advocate for optimal health practices among mothers and the community, to promote safe motherhood, good child health and appropriate family planning practices[19]. Households were selected with a sampling interval of two which was calculated from the list of 613 eligible households. Only one eligible participant was selected from each household. Therefore, the total number of households in the study was equal to the total number of mothers who were selected for the study. If more than one eligible participant was present in the same household then only one participant was selected using a lottery method. A total of twelve participants refused to provide complete information during the survey and thus, information from a total of 302 participants was included in the final analysis.

### Study instruments and variables

A semi-structured questionnaire was prepared by reviewing relevant literature including government policy documents and WHO guidelines. The survey questionnaire was arranged into two sections. The first section recorded the socio-demographic characteristics of participants and the second section collected information on mothers’ knowledge and practices of essential newborn care. Socio-demographic characteristics were captured through a structured questionnaire. The information collected in this section of the questionnaire included participants’ age, age at marriage, age at first pregnancy, ethnic group, education level, family type (nuclear or joint family), number of children, age of the youngest child, household monthly income, main sources of income, distance to health facility, maternal history of antenatal check-up, postnatal check-up for mother and baby, place of last delivery, maternal tobacco and alcohol consumption.

A semi-structured questionnaire was developed to assess a mother’s knowledge regarding newborn care and practices. The questions concerning newborn care knowledge and practices were developed based on WHO guidelines for essential newborn care. A total of 14 knowledge related questions were asked to respondents to assess the level of newborn care knowledge of mothers. The knowledge assessment questionnaire encompassed mother’s knowledge regarding the neonatal period, essential components of newborn care, recognition of neonate danger signs, causes of danger signs, safe cord caring practice, neonatal eye infection, early initiation of breastfeeding, exclusive breastfeeding, neonatal sleep patterns, neonatal vaccines, minimum number of neonate postnatal check-up, and Kangaroo Mother Care (KMC) for low birth weight neonates. A total of 10 questions were asked to mothers in order to assess newborn care practices. The newborn care practices questionnaire collected information on colostrum feeding, initiation of breastfeeding within one hour of birth, immediate wiping and wrapping of baby with clean, soft cloths, umbilical cord cutting practice, cord caring, skin to skin contact, delayed bathing, minimum number of postnatal check-up, received neonatal vaccination and exclusive breastfeeding.

To ensure the quality of data, the English version of the questionnaire was translated into Nepali language and the questionnaires were then pretested in Thuli Bheri Municipality of Dolpa district. Further corrections and required modifications of the pretested questionnaires were done prior to implementation of the survey.

### Data collection

Face to face interviews were conducted in the respondents’ households. Participants were interviewed using semi-structured, pre-tested questionnaires. The data were collected by five undergraduate public health students, who were fluent in Nepali language, were local residents of the survey district, and were involved in the study from the initial planning through to the data collection period.

The interviewers were also provided with three days of training on application of tools, sample selection, ethical considerations and techniques of data collection prior to conducting the survey. The average duration of each interview was approximately 45 minutes, resulting in four completed interviews per day for each enumerator over a period of sixteen days. The collected data were reviewed and validated daily to ensure the completeness and quality.

### Ethical statements

The study was conducted only after obtaining ethical clearance from the Ethical Review Board (ERB) of the Nepal Health Research Council (ERB Reg. no: 1072/2017) and District Health office of Dolpa. All the participants were informed about the purpose of the study and verbal with written informed consent was obtained from every participant prior to the interview. Informed consent documents were read to the participants and their literate representative in the case of illiterate participants. After verbal informed consent, participants and their representative’ signatures were obtained on the consent form. Collected data was anonymized and the information obtained from the participants was kept strictly confidential.

### Statistical analysis

The data were entered into EpiData software v3.1 and transferred into IBM SPSS 21 (Chicago, IL, USA) for statistical analysis. A score method was applied to analyze the responses of knowledge related questions. Every correct answer (consistent with WHO essential newborn care guidelines) received a score of 1 mark and an incorrect answer (inconsistent with WHO essential newborn care guidelines) or a “don’t know” response received a score of 0. The mean score of the responses was calculated from a total of 14 newborn care knowledge related questions to use as a cut-off point to differentiate between mothers having adequate and inadequate newborn care knowledge. Mothers scoring equal to or above the average score were considered to have adequate knowledge and those scoring below the average score were considered to have an inadequate level of knowledge regarding newborn care[20]. Similarly, a total of 10 questions were asked to assess practice of newborn care where the mean score was 6.2. Considering mean score and Bloom’s cut off points[21,22], mothers who practiced at least 6 good practices out of 10 were considered to be implementing satisfactory practices of newborn care (i.e. ≥ 60% correct practices) and those who did not practiced at least 6 good practices were considered to be practicing an unsatisfactory level (i.e. < 60% correct practices) of newborn care. Descriptive statistics were performed for the outcome and all explanatory variables to explore the characteristics of the chosen sample. Bivariate analysis was conducted between the two outcome variables pertaining to maternal knowledge of newborn care and newborn practices and the explanatory variables of interest, using chi-square tests of association or t-tests.

Binary logistic regression was employed to examine presupposed associations between mothers’ newborn care knowledge and socio-demographic characteristics, such as respondents age, age at marriage, age at first pregnancy, family type, education status, family monthly income, ANC check up, PNC check up, place of last delivery, and distance to reach the nearest health facility. The regression analysis was performed with the following dichotomous dependent variable: adequate versus inadequate knowledge and satisfactory versus unsatisfactory newborn care practices. Hosmer-Lemeshow tests were used to measure the goodness of fit of the models. All the significant predictor variables with a statistically significant p-value (<0.05) in univariate logistic regression analyses were included in the multivariate logistic regression models. The final results were reported using adjusted odds ratio (AOR) with 95% confidence intervals (CI).

## Results

### Demographic and socio-economic characteristics

Table 1 presents the demographic and socio-economic characteristics of mothers compared by their level of newborn care knowledge. A total of 302 (96.18%) mothers were included in the final analysis from the total 314 interviewed mothers. A total of 12 (3.82%) mothers refused to provide complete information during the interview and thus they were excluded from the final analyses. The mean ages of the study participants and that of their youngest child were 24.0 (± 3.6 SD) years and 12.3 (± 7.5 SD) months respectively. Similarly, mothers’ mean age at marriage was 17.8 (± 2.2 SD) years and the mean age at first pregnancy was 19.1 (±2.1 SD) years. Only 50.7% of households had a combined income over $100 USD per month. The average family monthly income reported by the study subjects was $120.1 (± $68.9 SD) USD per month. The proportion of mothers who had never attended school was 31%, a further 34.1% had primary (grades 1–5) education, 20.5% had achieved secondary (grades 6–10) education and only 14.2% were found to have attained higher secondary (grades 11–12) or university level education. More than half of the study participants were from upper castes (50.7%) followed by Dalit (36.8%) and Janajati (12.6%). Agriculture/farming was the major occupation for the majority of the participants. The average walking distance to reach a health facility was 38.7 (±33.5 SD) minutes, as reported by the mothers.

**Table 1:** Demographic and socioeconomic characteristics of mothers by newborn knowledge category.

Variables	Total Sample N = 302	Newborn care knowledge		*P-value*
		Inadequate n = 147	Adequate n = 155	
	n(%)	n(%)	n(%)	
**Age category**				0.071
15–20	56 (18.5)	33(22.4)	23(14.8)	
21–25	132(43.7)	58(39.5)	74(47.7)	
26–30	99(32.8)	52(35.4)	47(30.3)	
31–35	15(5.0)	4(2.7)	11(7.1)	
**Mean ± SD**	24.0 ± 3.6	23.6±3.4	24.3±3.7	0.082^a^
**Ethnicity**				0.001*
Upper Caste	153(50.7)	58(39.5)	95(61.3)	
Janajati	38(12.6)	21(14.3)	17(11.0)	
Dalit	111(36.8)	68(46.3)	43(27.7)	
**Education status**				<0.001*
Never attended school	94 (31.1)	65(44.2)	29(18.7)	
Primary education	103(34.1)	53(36.1)	50(32.3)	
Secondary education	62(20.5)	18(12.2)	44(28.4)	
Higher secondary education and above	43(14.2)	11(7.5)	32(20.6)	
**Type of Family**				0.845
Nuclear	210 (69.5)	103(70.1)	107(69.0)	
Joint	92(30.5)	44(29.9)	48(31.0)	
**Occupation**				<0.001*
Business	34 (11.3)	3(2.0)	31(20.0)	
Service	8(2.6)	3(2.0)	5(3.2)	
Labor	32(10.6)	19(12.9)	13(8.4)	
Home maker	48(15.9)	24(16.3)	24(15.5)	
Agriculture	180(59.6)	98(66.7)	82(52.9)	
**Monthly income (USD)**				<0.001*
**≤ 100**	149(49.3)	91(61.9)	58(37.4)	
**>100**	153(50.7)	56(38.1)	97(62.6)	
**Mean ± SD ($)**	120.1 ± 68.9	103.2±60.0	136±72.9	<0.001^a^*
**Age at marriage (years)**				0.374
<15 Years	25(8.3)	12(8.2)	13(8.4)	
16–20	247(81.8)	124(84.4)	123(79.4)	
21–25	30(9.9)	11(7.5)	19(12.3)	
**(Mean ± SD)**	17.8± 2.2	17.3±1.9	18.3±2.3	<0.001^a^*
**Age at first pregnancy** **(Years)**				<0.001*
**15–19**	196(64.9)	121(82.3)	75(48.4)	
**20–24**	90(29.8)	20(13.6)	70(45.2)	
**25–29**	16(5.3)	6(4.1)	10(6.5)	
**Mean ± SD**	19.1±2.1	18.5±1.8	19.6±2.3	<0.001^a^*
**Age of last baby (months)** **Mean ± SD**	12.3± 7.5	12.3±7.2	12.3±7.8	0.964^a^
**Number of child**				<0.001*
One	104(34.4)	61(41.5)	43(27.7)	
Two	133(44.0)	47(32.0)	86(55.5)	
Three and above	65(21.5)	39(26.5)	26(16.8)	
**Antenatal care visit**				<0.001*
One	30(9.6)	18(12.2)	12(7.7)	
Two	56(18.5)	38(25.9)	18(11.6)	
Three	46(15.2)	32(21.8)	14(9.0)	
Four and above	170(56.3)	59(40.1)	111(71.6)	
**Postnatal care visit**				<0.001*
No visit	127(42.1)	91(61.9)	36(23.2)	
1–2 times	92(30.5)	29(19.7)	63(40.6)	
3 times and above	83(27.5)	27(18.4)	56(36.1)	
**Place of delivery**				0.001*
Home	180(59.6)	102(69.4)	78(50.3)	
Health facility	122(40.4)	45(30.6)	77(49.7)	
**Distance of heath facility** **(Minutes)**				<0.001*
<30	162(53.6)	63(42.9)	99(63.9)	
≥30–59	87(28.8)	44(29.9)	43(27.7)	
≥60	53(17.5)	40(27.2)	13(8.4)	
**Mean ± SD**	38.7±33.5	44.1±36.7	33.6±29.5	0.007^a^*
**Smoking**				0.893
No	227(75.2)	111(75.5)	116(74.8)	
Yes	75(24.8)	36(24.5)	39(25.2)	
**Alcohol intake habit**				0.097
No	164(54.3)	87(59.2)	77(49.7)	
Yes	138(45.7)	60(40.8)	78(50.3)	

^a^ *P-value* from independent t-test; and others are from Chi-Square test.

* *P-value* <0.05.

SD, standard deviation.

Only 56.3% of the interviewed mothers were found to have completed four Antenatal Care (ANC) check-ups and 27.5% of mothers had completed three Postnatal Care (PNC) check-ups during their last pregnancy. Moreover, only 40.4% of total mothers had delivered their last child in a health facility.

### Mothers’ newborns care knowledge and associated factors

The mean score of newborn care knowledge among the total sample was 7.36 (±2.88 SD) (Table 2). The maximum score obtained was 12.15 and the minimum score was 2.00 out of a total 14 points. Considering the mean score of knowledge as the assigned cut-off point, 48.7% of mothers were found to have an inadequate level of newborn care knowledge and 51% of mothers were found to have an adequate level of newborn care knowledge. Further, chi-square and t-tests were applied where necessary to observe the significant associations between socio-demographic characteristics and maternal level of knowledge regarding newborn care.

**Table 2:** Newborn care knowledge among mothers and newborn care practices in upper Himalaya.

Newborn care knowledge			Correct response n (%)
Knowledge about newborn or neonate period			155(51.3)
Knowledge on at least 4 major component of immediate essential newborn care (immediate wipe and warping baby, hygienic cord cutting practice, skin to skin contact of baby and early initiation of breast feeding to newborn).			132(43.7)*
Knowledge on least 4 benefits of newborn essential care (prevents newborn from hypothermia, hypoglycemia, infections and pneumonia).			66(21.9)*
Knowledge on at least 5 major danger neonatal signs (Neonate unable to feed, unconsciousness, severe chest in drawing, convulsion, and fast breathing)			80(26.5)*
Knowledge on at least 5 causes of neonate danger signs. (Hypothermia, birth asphyxia, low birth weight, neonatal jaundice, and infections).			101(33.4)*
Knowledge on safe cord cutting practice			146(48.3)
Knowledge on cord caring practice (i.e. use of chlorexidine gel)			247(81.8)
Knowledge on at least 3 neonate eye infection danger signs (eye discharge, reddening of eye and swollen eye).			156(51.7)*
Knowledge on exclusive breast-feeding practice			214(70.9)
Knowledge of frequency of breast-feeding per day for newborn.			56(18.5)
Knowledge on requirement of minimum sleep hours for newborn.			89(29.5)
Knowledge on neonatal vaccine			258(85.4)
Knowledge on minimum number of postnatal checks ups for newborn.			76(25.2)
Knowledge on caring low birth weight newborn care. (Kangaroo mother care, early and frequent breast feeding and frequent consultation with clinician).			159(52.6)
Mean knowledge score (Mean ± SD) = 7.36 ±2.88			
Level of newborn care Knowledge	Inadequate (<mean knowledge score)		147(48.7)
	Adequate (≥ mean knowledge score)		155(51.3)
**Newborn care practices**			
Colostrum feeding			235(77.8)
Initiation of breast feeding within one hour			203(67.2)
**Umbilical Cord cutting practice**			
New/sterile blade used from hygienic delivery kit			259(85.8)
Sickle (*Hasiya*)			41(13.6)
Used non sterile blade			2 (0.7)
**Cord caring practice**			
Chlorhexidine gel/antibiotic ointment applied to cord stump			189(62.2)
Butter/Oil (cooking oil)			45(14.9)
Turmeric powder			36(11.9)
Do not apply anything			32(10.6)
Wrapping newborn with soft and clean cloths/towel			262(86.8)
Practice of skin to skin contact			116(38.4)
Practice of delayed bathing (after 24 hours)			140(46.4)
Baby had at least 3 PNC checks up completed			83(27.5)
Newborn received BCG vaccine			298(98.7)
Practice of exclusive breast feeding			164(54.3)
Level of newborn care practice		Unsatisfactory practices (<60% correct practices)	102(33.8)
		Satisfactory practices (≥60% correct practices)	200(66.2)

* Multiple responses

Results of the univariate regression analyses (Table 3) suggested that mothers’ level of newborn care knowledge was significantly associated with mothers’ age at first pregnancy, ethnic group, education status, occupation, family monthly income, number of children, ANC visit, PNC visit, place of last delivery and distance to nearest health facility. However, the multivariate regression model (Table 3) revealed that mothers who were 20–24 years old at first pregnancy were three times more likely to demonstrate an adequate level of knowledge of newborn care (AOR 3.89 at 95% CI 1.81–8.37), compared to mothers who first became pregnant before 20 years of age. Adequate maternal newborn knowledge was also found to be higher among women who had attended school and the strength of this association was greatest for women who completed at least higher secondary education (AOR = 5.92 at 95% CI 1.81–19.33).

**Table 3:** Factors associated with adequate maternal newborn care knowledge among mothers with child less than two years in Dolpa district, Nepal.

Variables	UOR (95%CI)	AOR (95%CI)
**Age at marriage (Years)**		
<15 Years	**1**	**-**
16–20	0.91(0.40–2.08)	**-**
21–25	1.59(0.54–4.69)	**-**
**Age at first pregnancy (Years)**		
15–19	**1**	**1**
20–24	5.64(3.17–10.02)*	3.89(1.81–8.37)*
25–29	2.68(0.93–7.70)	2.07(0.48–8.88)
**Ethnicity**		
Upper Caste	1.28(0.60–2.69)	0.96(0.30–3.05)
Janajati	2.59(1.56–4.28)*	2.34(1.13–4.84)*
Dalit	**1**	**1**
**Education status**		
Never attended school	**1**	**1**
Primary education	2.15(1.17–3.79)*	2.16(0.99–4.68)
Secondary education	5.47(2.71–11.05)*	4.93(1.82–13.33)*
Higher secondary education and above and above	6.52(2.89–14.70)*	5.92(1.81–19.33)
**Occupation**		
Business	**1**	**1**
Service	0.16(0.25–1.03)	0.10(0.00–1.43)
Labor	0.06(0.01–0.26)*	0.22(003–1.45)
Home maker	0.09(0.02–0.36)*	0.05(0.01–0.322)*
Agriculture	0.08(0.02–0.27)*	0.12(0.02–0.59)
**Monthly income in $ USD**		
≤ 100	**1**	**1**
>100	2.71(1.70–4.32)*	1.09(0.57–2.10)
**No. of child**		
One	**1**	**1**
Two	2.59(1.53–4.40)*	2.65(1.26–5.56)*
Three and above	0.94(0.50–1.77)	2.27(0.92–5.59)
**Antenatal care visit**		
One	**1**	**1**
Two	0.71(0.283–1.78) )	0.82(0.25–2.70)
Three	0.65(0.25–1.72)	0.61(0.17–2.09)
Four and above	2.82(1.27–6.25)*	2.89(1.04–7.96)*
**Postnatal care visit**		
No visit	**1**	**1**
1–2 times	5.49(3.05–9.85)* 000000000 0 0 0 0	1.60(0.68–3.74)
3 times and above	5.24(2.87–9.55)* 92.73–14.68)	2.79(1.16–6.72)*
**Place of delivery**		
Home	**1**	**1**
Health facility	2.23(1.39–3.58)*	0.81(0.36–1.83)
**Distance of heath facility (minutes)**		
<30	4.83(2.39–9.74)*	2.60(0.95–7.07)
≥30–59	3.00(1.41–6.39)*	3.66(1.43–9.33)*
≥60	**1**	**1**

* P-value <0.05

UOR, Unadjusted Odds Ratio.

AOR, Adjusted Odds Ratio.

**1** = Reference.

Mothers who had more than one child were more likely to have adequate knowledge, compared to mothers with only one child: mothers with a second child (AOR 2.65 at 95% CI 1.26–5.56), mothers with three or more children (AOR 2.27 at 95% CI 0.92–5.59). In terms of the health care utilization, mothers who had completed four ANC visits (AOR 2.89 at 95% CI 1.04–7.96) or three PNC visit (AOR 2.79 at 95% CI 1.16–6.72) during their last pregnancy were two times more likely to possess an adequate level of newborn care knowledge, compared to the mothers who had not completed four ANC or three PNC visits during their last pregnancy. Moreover, mothers whose nearest health facility was within a walking distance of 30–59 minutes were more than three times as likely to demonstrate adequate newborn care knowledge (AOR 3.66 at 95% CI 1.43–9.33), compared to those who had to walk for more than an hour in order to reach their nearest health facility.

### Newborn care practices and associated factors

The study showed that 200 (66.2%) of mothers had practiced at least six out of 10 good newborn care practices (satisfactory practices) and 102 (33.8%) of mothers had practiced less than six out of 10 good newborn care practices (unsatisfactory practices) (Table 2). In a univariate analyses (Table 4), the independent variables observed to have a significant association with mothers’ satisfactory level of practice were mothers age, age at first pregnancy, education status, monthly family income, number of children, distance to nearest health facility, ANC visit, PNC visit, place of last delivery, and newborn care knowledge. However, the multivariate logistic regression model (Table 4) revealed that only mother’s monthly family income, PNC visit and newborn care knowledge were significant predictors of satisfactory level of newborn care practices. Mothers whose family income was more than $100 USD per month (AOR 4.17 at 95% CI 1.75–9.69), completed three postnatal visits (AOR 3.27 at 95% CI 1.16–9.20) and had adequate newborn care knowledge (AOR 15.35 at 95% CI 5.82–40.47) were found to be more likely to practice satisfactory level of newborn care practices.

**Table 4:** Factors associated with adequate maternal newborn care practice among mothers with child less than two years in Dolpa district, Nepal.

Variables	Level of practices		UOR (95%CI)	AOR (95%CI)
	Unsatisfactory practices n(%)	Satisfactory practices n(%)		
**Age of respondent** **(Years)**				
15–20	26(25.5)	30(15.0)	1	1
21–25	36(35.3)	96(48.0)	2.31(1.20–4.42)*	2.65(0.83–8.48)
26–30	34(33.3)	65(32.5)	1.65(0.84–3.23)	1.50(0.40–5.59)
31–35	6(5.9)	9(4.5)	1.30(0.40–4.14)	0.68(0.67–6.96)
**Age at marriage** **(Years)**				
≤ 15	11(10.8)	14(7.0)	1	-
16–20	79(77.5)	168(84.0)	1.67(0.72–3.84)	-
21–25	12(11.8)	18(9.0)	1.17(0.40–3.45)	-
**Age at first pregnancy** **(Years)**				
15–19	76(74.5)	120(60.0)	1	1
20–24	19(18.6)	71(35.5)	2.36(1.32–4.23)*	0.61(0.21–1.73)
25–29	7(6.9)	9(4.5)	0.81(0.29–2.27)	0.72(0.08–5.95)
**Ethnicity**				
Upper caste	51(50.0)	102(51.0)	1	-
Janajati	12(11.8)	26(13.0)	1.08(0.50–2.32)	-
Dalit	39(38.2)	72(36.0)	0.92(0.55–1.54)	-
**Education status**				
Never attended school	46(45.1)	48(24.0)	1	1
Primary	39(38.2)	64(32.0)	1.57(0.89–2.77)	1.15(0.47–2.81)
Secondary	10(9.8)	52(26.0)	4.98(2.26–10.96)*	1.91(0.54–6.77)
Higher secondary and above	7(6.9)	36(18.0)	4.92(1.99–12.18)*	1.95(0.46–8.15)
**Family income (USD)**				
≤ 100	79(77.5)	70(35.0)	1	1
>100	23(22.5)	130(65.0)	6.37(3.8–11.03)*	4.17(1.75–9.69)*
**No. of child**				
One	43(42.2)	61(30.5)	1	1
Two	33(32.4)	100(50.0)	12.13(1.22–3.71)*	1.04(0.37–2.90)
Three and more	26(25.5)	39(19.5)	1.05(0.56–1.98)	1.34(0.37–4.78)
**Distance to health facility (minutes)**				
<30	42(41.2)	120(60.0)	3.45(1.81–6.58)*	2.05(0.65–6.42)
30–59	31(30.4)	56(28.0)	2.18(1.08–4.38)*	1.68(0.58–4.85)
≥ 60	29(28.4)	24(12.0)	1	1
**Antenatal care visit**				
One	15(14.7)	15(7.5)	1	1
Two	26(25.5)	30(15.0)	1.15(0.47–2.80)	1.83(0.46–7.21)
Three	22(21.6)	24(12.0)	1.09(0.43–2.73)	1.69(0.422–6.82)
Four and above	39(38.2)	131(65.5)	3.35(1.50–7.47)*	2.01(0.62–6.51)
**Postnatal care visit**				
No visit	64(62.7)	34(17.0)	1	1
1–2 times	21(20.6)	100(50.0)	8.96(4.78–16.79)*	2.61(0,966–7.06)
3 times and above	17(16.7)	66(330)	7.30(371–14.37)*	3.27(1.16–9.20)*
**Place of delivery**				
Home	97(95.1)	83(41.5)	1	1
Health facility	5(4.9)	117(58.5)	27.34(10.66–70.13)*	18.73(5.53–63.46)
**Knowledge of newborn care**				
Inadequate	85(83.3)	62(31.0)	1	1
Adequate	17(16.7)	138(69.0)	11.12(6.10–20.29)*	15.35(5.82–40.47)*

* P-value <0.05

UOR, Unadjusted Odds Ratio.

AOR, Adjusted Odds Ratio.

1 = Reference.

## Discussion

This study aimed to examine factors associated with knowledge of newborn care and newborn care practices in the remote upper Himalayas of Nepal. The main findings from this study showed that more than half (51.3%) of the mothers demonstrated an adequate level of newborn care knowledge. Similarly, a study conducted in Ethiopia found that 36.1% of postnatal mothers had adequate knowledge of newborn care with a 75% cut-off point [23], whereas another study from Ethiopia showed 80.4% of mothers had good knowledge on essential newborn care with more than 50% correct answers[24]. The essential newborn care knowledge demonstrated by mothers in the current study was relatively low and this is perhaps due to differences in the study setting as respondents in our study were from the most disadvantaged geographical location with high illiteracy and limited accessibility to health resources. Furthermore, the quality of the ANC services and its availability at the local health facilities may be significant influential factors for the level of mothers’ knowledge, as all the mothers reported at least one ANC visit.

Mothers’ health related knowledge, attitudes, and behaviour are highly influenced by level of education [25]. In the present study, adequate newborn care knowledge of mothers was found to be significantly associated with maternal education, occupation, ANC visit, PNC visit, household income, age at first pregnancy, and distance of health facility. These results are comparable to results from studies conducted in Kenya and Ethiopia, where mothers’ education, ANC and PNC visits were positively associated with knowledge of newborn care [23,26]

In our study, adequate newborn care knowledge was found to be better among mothers who had at least primary level education, with increased odds among those with secondary level education and higher. The mean age at marriage for women in this sample was 17.8 years and nationally representative surveys also suggest a similar median age at marriage[9]. Approximately 65% of mothers in this study reported that their first pregnancy occurred during their adolescent years (15–19 years). This result presents an estimate which is more than three times higher than the national estimate of adolescent pregnancy[9]. The majority of mothers in this study visited a health facility for an ANC check-up, however, nearly half of deliveries took place at home with limited assistance from skilled health workers. This result was consistent with the Nepal Demographic and Health Survey.

In the current study, the mothers who had attended four or and more ANC visits and three or more PNC visit were found more than twice more likely to have adequate newborn care knowledge. This may be due to the dissemination of recommended newborn care related information adequately disseminated to mothers during ANC and PNC visits[27]. Moreover, multiple visits perhaps has the potential to increase exposure of mothers to this information, resulting in improved levels of understanding. Distance to health facilities also plays a crucial role in utilization of maternal and neonatal services[28,29]. A study conducted at peripheral health facilities of Nepal revealed mothers who lived less than an hour from a health facility were more satisfied with maternal health services[28] which is in accordance to the results of this study. Mothers who lived less than one hour away from a health facility were more likely to have adequate knowledge and report satisfactory practices in this current study. Therefore, the assumption may be made that mothers living nearer to a health facility (< 1 hour walking distance) may be more likely to access healthcare for minor illness during pregnancy, as well as the postnatal period. Providing mothers with opportunities to easily access health personnel, improves overall health and knowledge regarding neonatal care.

The study revealed an overall satisfactory level of newborn care practice, which was 66.2%. However, less than half of total mothers had practiced delayed bathing of the newborn (after 24 hours of birth), skin to skin contact, immediate wrapping of newborn with clean and soft cloths, and at least three PNC check-ups. Furthermore, results from NDHS 2016 showed only one in five newborns (21%) received postnatal check-ups within the first hour of life, 75% of newborns received a postnatal check within the first 2 days following birth and the proportion who received three complete postnatal checks was very low[9]. With the aim of preventing neonatal health complications, the Government of Nepal recommends at least three postnatal checkups for newborns within 7 days of delivery, however results from both the present study and findings from NDHS 2016 suggest that further improvements need to be made in order to increase the number of postnatal check-ups that newborns receive. [9].

The Government of Nepal has promoted institutional delivery to prevent neonatal mortality and morbidity[30]. In our study, only 40.4% of last deliveries were conducted in a health facility. Many studies conducted in Nepal justified that the low institutional delivery rate was due to transportation inaccessibility, high additional indirect costs associated with institutional delivery and the longstanding tradition of home delivery [31,32]. In addition, 14.0% of newborns had their cord cut with a Sickle (*Hasiya*) and used non sterile blade. These traditional methods of cord cutting and caring practices have been reported as major risk factors for cord infection and neonatal tetanus [33,34]. Likewise, 62.2% had practiced safe cord caring practice (used chlorexidine in cord stump), whereas nearly 26% of mothers had applied butter/cooking oil/mustard oil/turmeric powder on the cord stump and 10.6% had applied nothing to the cord stump. In order to prevent the development of omphalitis which is a significant risk factor for neonatal mortality, it is vital that timely cord cleansing occurs with an appropriate antiseptic solution such as chlorhexidine[35]. Furthermore, prevention of infection at the site of the umbilical cord is particularly important in contexts such as rural Nepal where the symptoms of infection may develop unrecognised[35]. Approximately 67.2% of newborn were breastfed within an hour of birth. These results are comparable with the NDHS 2016 and similar studies conducted in rural Nepal [9,12,36]. Several studies in Nepal showed the reasons for poor cord cutting and caring practice were mainly due to deep rooted cultural belief, low awareness regarding cord caring antibiotic ointment (Chlorexidine gel), low maternal education, inability to perceive risks of poor neonatal care practices and less awareness about hygienic delivery kit or difficulty in obtaining the kit locally [12,36,37].

Furthermore, good newborn care practices were more than three folds higher among mothers who had a household monthly income over $100 USD and those who have completed at least three postnatal visits. Supporting this finding, studies conducted in Ethiopia and Pakistan also reported association between good newborn care practices and mothers’ newborn care knowledge and level of wealth [23,38,39]. In contrast, a study conducted by Misgna et al. showed no association between newborn care practices and income status [24]. The differences are perhaps due to differences in modality of the health care delivery system, availability of health services and awareness strategies for maternal and child health in different countries[24]. Likewise, the probability of good practice was more than fifteen times higher among mothers who had adequate newborn care knowledge. The result is comparable with a study conducted in Ethiopia in which mothers who had adequate newborn care knowledge were more likely to demonstrate good practice [24].

While this study advances our knowledge in the area of newborn care knowledge and practices, it is not without limitations. First, the results regarding the practice of newborn care are based on mothers’ recall. To minimize the recall bias, the interview was only limited to mothers with children under two years. Since the study was of a cross-sectional design, predication of strong causal relationships was not possible. Some of the results with wide confidence intervals due to the small sample size, may limit the precise estimates of the strength of association. Similarly, it was not feasible to observe the newborn care practices and so, the information was solicited from mothers through use of questionnaires, which may lead to social desirability bias. Despite these limitations, the study has explored the maternal newborn care knowledge and newborn care practices in the upper Himalayas that has been not attempted by earlier studies. The findings of this study can play a crucial role in planning interventions, particularly for districts in the mountainous region of Nepal and similar settings. Additionally, this study has also set the background for further qualitative studies to further understanding and explore the possibilities of discouraging traditional beliefs and cultural practices of newborn care that are associated with high risks of neonatal morbidity and mortality in this region. Likewise, it can be suggested that there should be further exploration of the linkages between newborn outcomes and knowledge and practices within contexts of poverty, gender disparity and vast geographical variation. In line with the 2016 United Nations Sustainable Development Agenda of “leaving no one behind”, this study focuses on a sub-population in a hard-to-reach district where there is limited data availability to ensure that those who are most disadvantaged are also represented[40].

## Conclusion

The study revealed a high prevalence of inadequate newborn care knowledge and almost one third of mothers practiced poor newborn care in upper Himalayan dwellings. The results of our study suggest that increased frequency of ANC and PNC visits likely enhances the knowledge and practices of newborn care. Promotion of institutional delivery and postnatal visits both for both mothers and newborns needs to be encouraged through the telemonitoring system as well as regular follow-up visits at home for neonatal care, in additional to the current safe motherhood scheme. Awareness of safe cord cutting and caring practices needs to be disseminated. Since a significant number of mothers had reported long walking distances to reach a health facility as a major hindering factor in maternal and neonatal service utilization, the local government should invest in universal health coverage to increase the accessibility of health services for mothers and newborns.

## Supporting information

S1 File Raw data set. (SAV)

S2 File English version of tools. (DOCX)

S3 File Nepali version of tools. (DOCX)

## Abbreviations

ANC: Antenatal Care
AOR: Adjusted Odds Ratio
CB-NCP: Community-Based Newborn Care Program
CBS: Center Bureau of Statistics
COR: Crude Odds Ratio
FCHVs: Female Community Health Volunteers
PNC: Postnatal Care
SDGs: Sustainable Development Goals
