# Supplementary material for: Factors associated with newborn care knowledge and practices in the upper Himalayas
Source: PLoS One. 2019 Sep 16;14(9):e0222582. doi: 10.1371/journal.pone.0222582 (PMC6746396; doi:10.1371/journal.pone.0222582)
Supplement: S2 File — (DOCX) [file pone.0222582.s002.docx]

| **Part-1 Socio demographic characteristics of mothers having child younger than 24 months** | | | |
| --- | --- | --- | --- |
| **No.** | **Question** | **Response** | **Skip** |
| 101 | Caste/ethnicity of the respondent: |  |  |
| 102 | Age of respondent (years): |  |  |
| 103 | Respondent age at Marriage (years): |  |  |
| 104 | Age of respondent during first pregnancy(years): |  |  |
| 105 | Age of last child ( months) : |  |  |
| 106 | Sex of last child: | 1. Male |  |
|  |  | 1. Female |  |
| 107 | Type of family: | 1. Nuclear |  |
|  |  | 1. Joint |  |
| 108 | Total live number of children: |  |  |
| 109 | Total number of family member: |  |  |
|  | Children (below 18 years) |  |  |
|  | Adult |  |  |
| 110 | Education status of respondent : | 1. No education |  |
|  |  | 1. Primary education |  |
|  |  | 1. Secondary education |  |
|  |  | 1. Higher secondary education |  |
|  |  | 1. University education |  |
| 111 | Occupation of respondent : | Agriculture |  |
|  |  | Business |  |
|  |  | Services (Public or Private) |  |
|  |  | Home makers |  |
|  |  | Wage based Labor/manual work |  |
|  |  | Others (specify)……………………… |  |
| 112 | Family monthly income (NRS): |  |  |
| 113 | Distance to reach nearest health facility: |  |  |
|  | Walking distance: | …………. hour ……………… minutes |  |
|  | By vehicle: | …………….. hour………… minutes |  |
| 114 | Place of delivery of last child: | Home |  |
|  |  | Health facility |  |
| 115 | Have you visited health facility for ANC checkup during your last pregnancy? | 1. Yes |  |
|  |  | 1. No | Go to Q. no. 117 |
| 116 | If yes, how many times did you had ANC checkup during your last pregnancy? | One time |  |
|  |  | Two times |  |
|  |  | Three times |  |
|  |  | Four times |  |
|  |  | More than four times |  |
| 117 | Do you had Post-natal check-up during last delivery? | Yes |  |
|  |  | No | Go to Q. no.119 |
| 118 | If yes, how many times do you had PNC checkup during your last delivery? | One time check up |  |
|  |  | Two check up |  |
|  |  | Three check up |  |
|  |  | More than three check up |  |
| 119 | Smoking behavior of respondent | Yes |  |
|  |  | No |  |
| 120 | Alcoholic consumption respondent | Yes |  |
|  |  | No |  |

| **Part -2 Knowledge on Newborn care among mothers having child younger than 24 months** | | | |
| --- | --- | --- | --- |
| **No.** | **Question** | **Response** | **Skip** |
| 201 | What is the period of neonate? | ……………………….. days |  |
|  |  | Don’t know |  |
| 202 | What are the essential newborn cares?  (Multiple responses) | Immediate wipe and warping baby |  |
|  |  | Hygienic cord cutting practice |  |
|  |  | Early initiation of breast feeding to newborn |  |
|  |  | Place the baby in skin to skin contact |  |
|  |  | Don’t know |  |
|  |  | Others (specify)……………… |  |
| 203 | What are major benefits of essential newborn care to baby? (Multiple responses) | Prevents newborn from hypothermia |  |
|  |  | Prevents newborn from hypoglycemia |  |
|  |  | Prevents newborn from Infections |  |
|  |  | Prevents newborn from pneumonia |  |
|  |  | Don’t know |  |
|  |  | Others (specify)…………………. |  |
| 204 | What are major neonate danger signs? (Multiple responses) | Neonate unable to feed |  |
|  |  | unconsciousness, |  |
|  |  | severe chest in drawing |  |
|  |  | convulsion |  |
|  |  | fast breathing |  |
|  |  | Don’t know |  |
|  |  | Others (specify)……………….. |  |
| 205 | What are major causes of neonate danger signs? (Multiple responses) | Hypothermia |  |
|  |  | birth asphyxia |  |
|  |  | low birth weight |  |
|  |  | neonatal jaundice |  |
|  |  | infections |  |
|  |  | Don’t know |  |
|  |  | Others (specify)…………. |  |
| 206 | What material are safe to use to cut umbilical cord? | Sterile blade |  |
|  |  | Don’t know |  |
|  |  | Others (specify)…………. |  |
| 207 | What should be applied to umbilical stump after it cut? | Chlorhexidine gel/antibiotic ointment applied to cord stump |  |
|  |  | Butter/Oil (cooking oil) |  |
|  |  | Turmeric powder |  |
|  |  | Do not apply anything |  |
|  |  | Others (specify) |  |
| 208 | What are neonate eye infection danger signs? (Multiple responses) | eye discharge |  |
|  |  | reddening of eye |  |
|  |  | swollen eye |  |
|  |  | Others (specify) |  |
| 209 | Do you have knowledge regarding when to start the first breast feeding? | Yes |  |
|  |  | No | Go to Q.no.211 |
| 210 | If yes, please mention the time to start the first breast feeding after birth. | 1. Within an hour of birth 2. After an hour of birth |  |
| 211 | Do you have information regarding how many times newborn should breast fed in 24 hours? | Yes |  |
|  |  | No | Go to Q. no.213 |
| 212 | If yes, please mention how many times newborn should breast fed within 24 hours? | 1. 8 times a day and 4 times a night 2. Don’t know 3. Others |  |
| 213 | Do you have information regarding exclusive breast feeding? | Yes |  |
|  |  | No | Go to Q.no.215 |
| 214 | If yes, what is the period of exclusive breast feeding? | 6 months |  |
|  |  | Don’t know |  |
|  |  | Others ………. |  |
| 215 | Do you have information regarding minimum sleep time for newborn baby? | Yes |  |
|  |  | No | Go to Q.no.217 |
| 216 | If yes, what is the minimum sleep time for newborn baby? | a)……………  b) Don’t know |  |
| 217 | What vaccine are given to newborn baby in the first month of birth? | BCG |  |
|  |  | DPT-HepB-HiB |  |
|  |  | Oral Polio vaccine |  |
|  |  | Pneumococcal conjugate vaccine |  |
|  |  | Measles-Rubella |  |
|  |  | Japanese Encephalitis |  |
|  |  | Others (specify ) |  |
| 218 | What is the minimum times of postnatal visit of newborn baby? | ……………………. |  |
| 219 | Do you have knowledge regarding care of low birth weight newborn? | Yes |  |
|  |  | No | Go to Q.no.301 |
| 220 | If yes, what are the cares to low birth weight newborn? (Multiple responses) | Kangaroo mother care |  |
|  |  | early and frequent breast feeding |  |
|  |  | frequent consultation with clinician |  |
|  |  | Others (specify)……….. |  |

| **Part-3 Newborn care practices** | | | |
| --- | --- | --- | --- |
| **No.** | **Question** | **Response** | **Skip** |
| 301 | Where did you deliver you last child? | Home |  |
|  |  | Health facility |  |
| 302 | Did your child fed with colostrum, the first liquid that come from your breast? | Yes |  |
|  |  | No |  |
| 303 | What instrument was used to cut the umbilical cord of your newborn? | New/sterile blade from hygienic delivery kit |  |
|  |  | Sickle (Hasiya) |  |
|  |  | Used blade ( unsterile blade) |  |
|  |  | Others specify |  |
| 304 | Do you remember what was applied to the stump, when cord was cut? | Yes |  |
|  |  | No | Go to Q.no.306 |
| 305 | If yes, please mention the materials applied to the stump of your newborn? | Chlorhexidine gel/antibiotic ointment applied to cord stump |  |
|  |  | Butter/Oil (cooking oil) |  |
|  |  | Turmeric powder |  |
|  |  | Cow dung |  |
|  |  | Ash |  |
|  |  | Do not apply anything |  |
|  |  | Others( specify) |  |
| 306 | At what time after the birth, your baby was breast fed? | First one hours |  |
|  |  | After one hour |  |
| 307 | Did you give additional diet (pre-lacteal) within 28 days? | Yes |  |
|  |  | No |  |
| 308 | Did you fed your child only breast milk until 6 months? (if child is less than six month, ask mother if she is continuing exclusive breast feeding or not) | Yes |  |
|  |  | No |  |
| 309 | Do you remember, What was uses to wrap the baby immediately after birth? | Yes |  |
|  |  | No | Go to Q.no.311 |
| 310 | If yes, What was uses to wrap the baby immediately after birth? | Clean, soft cloths/towel |  |
|  |  | Used cloths |  |
|  |  | Others (specify) |  |
| 311 | Did you keep your newborn immediate skin to skin contact? | Yes |  |
|  |  | No |  |
| 312 | When did your baby first bath after birth? | First 24 Hours |  |
|  |  | After 24 hours |  |
| 313 | Did your baby had post natal check-up? | Yes |  |
|  |  | No | Go to Q.no.315 |
| 314 | If yes, how many times did your baby had postnatal check within first month of birth? | One time |  |
|  |  | Two times |  |
|  |  | Three times |  |
|  |  | More than three times |  |
| 315 | Did your child received vaccine in first month of the birth. | Yes |  |
|  |  | No | End the survey |
| 316 | If yes, what vaccine your child receive in the first month of birth? | BCG |  |
|  |  | DPT-HepB-HiB |  |
|  |  | Oral Polio vaccine |  |
|  |  | Pneumococcal conjugate vaccine |  |
|  |  | Measles-Rubella |  |
|  |  | Japanese Encephalitis |  |
|  |  | Others (specify ) |  |

Thank you for your valuable time and consideration.
