# Supplementary material for: Factors associated with newborn care knowledge and practices in the upper Himalayas
Source: PLoS One. 2019 Sep 16;14(9):e0222582. doi: 10.1371/journal.pone.0222582 (PMC6746396; doi:10.1371/journal.pone.0222582)
Supplement: S3 File — (DOCX) [file pone.0222582.s003.docx]

| **efu s @$ dlxgfeGbf sd pd]/sf aRrfx? ePsf cfdfx?sf] ;fdflhs tyf hg;f+lVos ljj/0f** | | | |
| --- | --- | --- | --- |
| k\|=g+= | k\|Zg | k\|ltls\|ofx? | s}lkmot |
| !)! | ;xefuLsf] hfltotf |  |  |
| !)@ | ;xefuLsf] pd]/ -jif{df_ |  |  |
| !)# | ;xefuLsf] ljjfx ubf{sf] pd]/ -jif{df_ |  |  |
| !)$ | klxnf] k6s ue{jtL x'bfsf] pd]/ -jif{df_ |  |  |
| !)% | xfn;fn} hlGdPsf] jf kl5Nnf] aRrfsf] pd]/ -महिनाdf_ |  |  |
| !)^ | xfn;fn} hlGdPsf] jf kl5Nnf] aRrfsf] lnª\u | s_ k'?if  v_ dlxnf |  |
| !)& | kl/jf/sf] lsl;d | s_ Psn  v_ ;+o'Qm |  |
| !)* | s'n lhljt aRrfx?sf] ;ª\Vof -!* jif{ d'lgsf_ |  |  |
| !)( | s'n kl/jf/ ;ª\Vof  aRrfx? -!* jif{ d'lgsf_ |  |  |
|  | jo:sx? |  |  |
| !!) | ;xefuLsf] z}lIfs of]Uotf | s_ cf}krfl/s lzIff gePsf]  v_ k\|fylds txsf] lZfIff  u_ dfWolds txsf] lzIff  3_ pRr–dfWolds txs]f lzIff  ª_ ljZj ljBffno txsf] lzIff |  |
| !!! | ;xefuLsf] k]zf | s_ s[lif  v_ Jofkf/  u_ ;]jf -lglh tyf ;/sf/L_  3_ u[x0fL  ª_ Hofnf dhb'/L  r_ cGo -v'nfpg'xf];_ |  |
| !!@ | kl/jf/sf] dfl;s cfDbfgL -g]=?=_ |  |  |
| !!# | glhssf] :jf:Yo ;+:yf ;Ddsf] b'/L  lx8]/ | =========306f  ==========ldg]6 |  |
|  | df]6/df | ==========306f  ========ldg]6 |  |
| 114 | kl5Nnf] aRrf hlGdPsf] 7fFp | s_ 3/  v_ :jf:Yo ;+:yf |  |
| !!% | s] tkfO{ kl5Nnf] rf]l6 ue{jlt x'Fbf ue{hfFrsf nflu :jf:Yo ;+:yfdf hfg'ePsf] lyof]< | s_ uPsf] lyP |  |
|  |  | v_ uPsf] lyPg | k\|=g+=!!& df hfg'xf];\ |
| !!^ | olb ue{hfrsf] nflu hfg'ePsf] lyof] eg], kl5Nnf] k6s ue{jlt x'Fbf tkfO{ slt k6s ;Dd ue{hfFrsf nflu hfg'eof]< | s_ Ps k6s  v_ b'O{ k6s  u_ ltg k6sp  3_ rf/ k6s  ª_ rf/ k6seGbf w]/} |  |
| !!& | s] tkfO{n] kl5Nnf] k6s ;'Ts]/L xF'bf, ;'Ts]/L eO;s] kZrft u/fpg] hfFr u/fpg'eof]< | s_ u/] |  |
|  |  | v_ ul/g | k\|=g+=!!( df hfg'xf];\ |
| !!* | olb ;'Ts]/L kZrft ul/g] hfFr u/fpg' eof] eg] slt k6s ;Dd u/fpg'eof]< | s_ Ps k6s  v_ b'O{ k6s  u_ tLg k6s  3_ tLg k6seGbf w]/} |  |
| !!( | ;xefuLsf] w'd\|kfg ug]{ afgL | s_ 5  v_ 5}g |  |
| !@) | ;xefuLsf] dBkfg ug]{ afgL | s_ 5  v_ 5}g |  |
| **efu v @$ dlxgfeGbf sd pd]/sf aRrfx? ePsf cfdfx?sf] gjhft lzz' :ofxf/ ;DalGw 1fg** | | | |
| @)! | slt ;do cjlw ;Dd lzz'nfO{ gjhft lzz' elgG5< | hGd]sf] ===============lbg |  |
| @)@ | gjhft lzz'sf cfjZos :ofxf/x¿ s'g s'g x'g< | s_ aRrfnfO{ t'¿Gt} k'5\g] / n'ufdf a]g]{  v_ gflenfO{ :jR5 tl/sfn] sf6\g] cEof;  u_ gjhftnfO{ t'?Gt} cfdfsf] ljuf}tL b'w r';fpg  3_ aRrfsf] 5fnfnfO{ cfdfsf] 5fnf;Fu hf]8\g]  ª_ cGo -v'nfpg'xf];_== |  |
| @)# | gjhft lzz'nfO{ cfjZos kg]{ :ofxf/x¿ k\|bfg u/] kZrft x'g] d'Vo kmfObfx¿ s]–s] x'g< (ax'pQ/) | s_ gjhft lzz'sf] z/L/ lr;f]af6 aRg]  v_ gjhftsf] zl//df Un'sf]hsf] dfqf sd x'gaf6 aRg]  u_ gjhft ;ª\s\|d0f x'gaf6 aRg]  3_ gjhft lgdf]lgof x'gaf6 aRg]  ª_ yfxf 5}g  r_ cGo -v'nfpg'xf];_ |  |
| @)$ | gjhftdf b]lvg] d'Vo vt/fsf ;ª\s]tx¿ s]–s] x'g< | s_ gjhftnfO{ b'w r':g ufx\|f] x'g]  v_ cr]t x'g]  u_ cToflws sf]vf xfGg]  3_ sfk]/ d'l5{t x'g]  ª_ l56f]l56f] Zjf; km]g]{  r_ yfxf 5}g  5_ cGo -v'nfpg'xf];_== |  |
| @)% | gjhftsf vt/fsf ;ª\s]t b]lvg'sf sf/0fx¿ s]–s] x'g< (ax'pQ/) | s_ s7\oflª\u\|g'  v_ hldGbf aRrfnfO{ Zjf; km]g{ ufx\|f] x'g'  u_ sd tf}nsf] aRrf hlGdg'  3_ gjhftnfO{ hlG8; x'g'  ª_ ;ª\s\|d0fx¿ x'g'  r_ yfxf 5}g  5_ cGo -v'nfpg'xf];_== |  |
| @)^ | gjhft lzz'sf] gfn sf6\gnfO{ s'g ;'/lIft ;fdfu\|L k\|of]u ug'{k5{ < | s_ hLjf0f' /lxt÷gofF kQL  v_ yfxf 5}g  u_ cGo -v'nfpg'xf];_ |  |
| @)& | Gffle sfl6;s] kl5 gfledf s] nufpg' k5{< | s_ gfle+ dnd  v_ gf}gL÷vfg]t]n  u_ a];f/  3_ s]lx klg gnufpg]  ª_ cGo -v'nfpg'xf];_== |  |
| @)* | gjhft lzz'sf cfFvf;Fu ;DalGwt vt/fsf lrGxx¿ s]–s] x'g\<(ax'pQ/) | s_ cfFvf r'lxg'  v_ cfFvf /ftf] x'g'  u_ cfFvf ;'lgg'  3_ cGo -v'nfpg'xf];_ |  |
| @)( | :tgkfg slxn] ;'¿jft ug'{k5{ eg]/ s] tkfOnfO{ yfxf 5< | s_ 5 |  |
|  |  | v_ 5}g | k\|=g+=@!! df hfg'xf];\ |
| @!) | olb yfxf 5 eg], s[kof at+fO{lbg'; aRrfnfO{ hGd]sf] slt ;do kl5 klxnf] :tgkfg u/fpg' k5{< | s_ klxnf] ! 306fdf v_ Ps 306f kZrft |  |
| @!! | gjhft lzz'nfO{ @$ 306fdf slt rf]6L :tgkfg u/fpg' k5{< | s_ yfxf 5 |  |
|  |  | v_ yfxf 5}g | k\|=g+=@!#df hfg'xf];\ |
| @!@ | olb yfxf 5 eg], s[kof atfOlbg'; gjhft lzz'nfO{ @$ 306fdf slt k6s :tgkfg u/fpg' k5{ | ===============k6s |  |
| @!# | k"0f{ :tgkfgsf] af/]df tkfO{nfO{ hfgsf/L 5< | s_ 5  v_ 5}g | k\|=g+=@!% df hfg'xf];\ |
| @!$ | olb yfxf 5 eg], k"0f{ :tgkfgsf] cjlw slt ;dosf] x'G5< | s_ 5 dlxgf  v_ yfxf 5}g  u_ cGo -v'nfpg'xf];_ |  |
| @!% | s] tkfOnfO{ gjhft lzz'nfO{ cfjZos kg]{ Go"gtd lgbfpg] ;dosf] af/]df yfxf 5< | s_ 5 |  |
|  |  | v_ 5}g | k\|=g+=@!& df hfg'xf];\ |
| @!^ | olb yfxf 5 eg], gjhft lzz'nfO{ Go""gtd slt ;dosf] lgGb\|f rflxG5< |  |  |
| @!& | gjhft lzz' hlGdPsf] klxnf] dlxgfdf s'g +vf]k nufOG5< | s_ la=;L=hL  v_ l8kL6L–x]k–aL–lxa  u_ cf]=lk=eL=  3_ l+gdf]sf]sn sGh'–u]6 vf]k  ª_ bfb'/f–¿a]nf  r_ hfklgh OG;kmnfOl6;  5_ cGo -v'nfpg'xf];_ |  |
| @!* | ;''Ts]/L kZrft gjhftnfO{ slt k6s ;Dd ;'Ts]/L kZrftsf] hfFr u/fpg'k5{< | ===================k6s |  |
| @!( | s] tkfOnfO{ hlGdb} sd tf}n ePsf] aRrfnfO{ s;/L :ofxf/ ug'{k5{ eGg] yfxf 5< | s_ 5 |  |
|  |  | v_ 5}g | k\|=g+=#)! df hfg'xf];\ |
| @@) | olb yfxf 5 eg], sd tf}n eP/ hlGdPsf] aRrfnfO{ s]–s] :ofxf/x¿ rflxG5g< (ax'pQ/) | s_ sª\uf¿ db/ s]o/  v_ lauf}lt b'w v'jfpg] / a]nf–a]nfdf :tgkfg u/fO/xg]  u_ lrlsT;saf6 lg/Gt/ k/fdz{ lnO{/fVg]  3_ cGo -v'nfpg'xf];_ |  |
|  |  |  |  |

**efu u gjhft lzz' :ofxf/ ;DalGw cEof;**

| #)! | tkfOn] kl5Nnf] aRrf sxfF hGdfpg' eof]< | s_ 3/d} v_ :jf:Yo ;+:yf |  |
| --- | --- | --- | --- |
| #)@ | s] tkfOn] cfkm\gf] aRrfnfO{ lauf}tL b'w v'jfpg' eof]< h'g ;'Ts]/L kZrft :tgaf6 klxnf] k6s cfp5 . | s_ v'jfP v_ v'jfOg |  |
| #)# | gjhft lzz'sf] gfle sf6\gsf nflu s] k\|of]u ug'{eof]< | s_ ;'Ts]/L emf]nfaf6 gof An]8  v_ xl;of  u_ k'/fgf] An]8 3_ cGo -v'nfpg'xf];_ |  |
| #)$ | s] tkfOnfO{ ofb 5, tkfOn] gfle sfl6;s] kl5 To;df s] nufpg' ePsf] lyof]< | s_ 5 v_ 5}g | k\|=g+=#)^ df hfg'xf];\ |
| #)% | olb ofb 5 eg], s] nufpg' ePsf] lyof]< | s_ gfle dnd v_ gf}gL÷vfg] t]n  u_ a];f/ 3_ ufO{sf] uf]a/  3_ v/fgL r_ s]lxklg nufOg 5_ cGo -v'nfpg'xf];_ |  |
| #)^ | aRrf hGd]sf] slt ;do kZrft lauf}tL b'w v'jfpg' eof]< | s_ klxnf] ! 306fdf v_ Ps 306f kZrft |  |
| #)& | s] tkfOn] aRrf hGd]sf] @* lbg leq cfdfsf] b'w cnfjf c¿ s]lx lrh aRrfnfO{ v'jfpg' eof]< | s_ v'jfP v_ v'jfOg===== |  |
| #)* | tkfO{n] cfkm\gf] aRrfnfO{ 5 dlxgf ;Dd cfkm\gf] b'w dfq v'jfpg'eof] -olb aRrf 5 dlxgf eGbf sd pd]/sf] 5 eg]], cfdfnfO{ ;f]Wg'xf];, s] pxfFn] aRrfnfO{ k"0f{ :tgkfg u/fO/fVg' ePsf] 5_< | s_ 5 v_ 5}g |  |
| #)( | s] tkfOnfO{ ofb 5, aRrf hGdg] lalQs} tkfOn] aRrfnfO{ s]n] a]g'{ePsf] lyof]< | s_ ofb 5 v_ ofb 5}g | k\|=g+=#!! df hfg'xf];\ |
| #!) | olb ofb 5 eg], aRrf hGdg] lalQs} s]n] a]g'{ ePsf] lyof]< | s_ ;kmf / g/d tf}lnofn] v_ k\|of]u u/]sf] n'ufx¿n]  u_ cGo -v'nfpg'xf];_ |  |
| #!! | s] tkfOn] aRrf hlGdg] lalQs} aRrfsf] zl// cfkm\gf] zl//df 6fF;]/ /fVg'eof]< | s_ /fv] v_ /flvg |  |
| #!@ | tkfOn] cfkm\gf] aRrf hlGdPsf] slt ;do kZrft p;nfO{ g'xfOlbgeof]< | s_ hlGdPsf] @$ 306f leq v_ hlGdPsf] @$ 306f kZrft |  |
| #!# | s] tkfOn] cfkm\gf] aRrf hlGd;s] kZrft ;'Ts]/L hfFr u/fpg' eof]< | s_ u/fP v_ u/fOg | k\|=g+=#!% df hfg'xf];\ |
| #!$ | olb u/fpg'eof] eg], hlGdPsf] Ps dlxgf leq aRrfnfO{ slt k6s ;Dd hFrfpg'eof]< | s_ Ps k6s v_ b'O{ k6s u_ ltg k6s  3_ ltg eGbf w]/} k6s |  |
| #!% | s] tkfOn] cfkm\gf] aRrfnfO{ hlGdPsf] Ps dlxgf leq s'g} vf]k nufpg'eof]< | s_ nufP v_ nufOg | ;e]{ ;dfKt |
| #!^ | olb nufpg'eof] eg], tkfOsf] aRrfnfO{ hlGdPsf] klxnf] dlxgfdf s'g vf]k nufpg'eof]< | s_ la=l;=lh= v_ l8lkl6–x]k aL–lxa  u_ cf]=lk=le=  3_ lgdf]sf]sn sGh'u]6 vf]k  ª_ bfb'/f–¿a]nf r_ hfklgh OlG;kmnfOl6;  5_ l66fg; l8ky]/Lof h_ cGo -v'nfpg'xf];_ |  |

tkfOsf] cd'No ljrf/ / ;dosf nflu wGojfb .
